# A tutorial on open-source large language models for behavioral science

**DOI:** 10.3758/s13428-024-02455-8

**Published:** 2024-08-15

**Authors:** Zak Hussain, Marcel Binz, Rui Mata, Dirk U. Wulff

**Affiliations:** 1 University of Basel, Basel, Switzerland; 2 Max Planck Institute for Human Development, Berlin, Germany; 3 Max Planck Institute for Biological Cybernetics, Tübingen, Germany; 4 Helmholtz Center for Computational Health, Neuherberg, Germany

**Keywords:** Large language models, Behavioral science, Hugging face

## Abstract

Large language models (LLMs) have the potential to revolutionize behavioral science by accelerating and improving the research cycle, from conceptualization to data analysis. Unlike closed-source solutions, open-source frameworks for LLMs can enable transparency, reproducibility, and adherence to data protection standards, which gives them a crucial advantage for use in behavioral science. To help researchers harness the promise of LLMs, this tutorial offers a primer on the open-source Hugging Face ecosystem and demonstrates several applications that advance conceptual and empirical work in behavioral science, including feature extraction, fine-tuning of models for prediction, and generation of behavioral responses. Executable code is made available at github.com/Zak-Hussain/LLM4BeSci.git. Finally, the tutorial discusses challenges faced by research with (open-source) LLMs related to interpretability and safety and offers a perspective on future research at the intersection of language modeling and behavioral science.

## Introduction

Large language models (LLMs) – machine learning systems trained on vast amounts of text and other inputs – are increasingly being used in science (Van Noorden & Perkel, 2023), and significantly advancing the capacity to analyze and generate meaningful linguistic information. These models are poised to change the scientific workflow in numerous ways and are already used across all aspects of the research cycle, from conceptualization to data analysis. For example, in psychology (Demszky et al., 2023) and related disciplines (Korinek, 2023), LLMs are being used to automate research processes, predict human judgments, and run in-silico behavioral experiments.

**Fig. 1:**
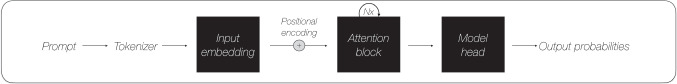
Overview of the LLM processing pipeline. The diagram illustrates the sequence of operations performed on an input prompt, including how it is tokenized and then processed by the transformer architecture to produce a set of output probabilities

Scientific applications of LLMs require high levels of transparency and reproducibility (Bockting et al., 2023). In addition, many applications in behavioral science involve sensitive information (e.g., personal or health data) or target vulnerable populations (e.g., children) and thus require specific data protection protocols. Open-source frameworks that provide full transparency and respect privacy requirements are therefore indispensable for applications of LLMs in behavioral science.

We aim to help advance the responsible use of LLMs in behavioral science by providing a comprehensive tutorial on applications using an open-source framework that maximizes transparency, reproducibility, and data privacy. Specifically, we provide a primer on the Hugging Face ecosystem, covering several applications of LLMs, including conceptual clarification, prediction of behavioral outcomes, and generation of human-like responses. Our target audience consists of behavioral researchers with a basic knowledge of programming principles who are interested in adding LLMs to their workflows. We hope that this resource will help researchers in psychology and related disciplines to adopt LLMs for a wide range of tasks, whilst also maintaining an appreciation of the subtle complexities of drawing scientific conclusions from such flexible and opaque models.

In what follows, we first provide a short primer on transformer-based language models. Second, we consider applications of LLMs in behavioral science and introduce the Hugging Face ecosystem and associated Python libraries. Third, we present three areas of application – feature extraction, fine-tuning, and text generation – and present several use cases in behavioral research. Finally, we address some advantages and limitations of current open-source approaches and consider future directions at the intersection of LLMs and behavioral research.

## A primer on transformer-based language models

Today’s LLMs are based on the transformer architecture (Vaswani et al., 2017), which is a family of neural network models that draw on a common set of building blocks. In this section, we first introduce these building blocks in sequence (see Fig. 1) before discussing important architectural variants and ways of applying LLMs in behavioral science.

### Tokenizers

When text input is fed into an LLM, it is first broken up into digestible pieces known as tokens. This decomposition is carried out by the LLM’s tokenizer, which is a model in its own right. For instance, the (uncased) *WordPiece* tokenizer (Wu et al., 2016) underlying the popular class of BERT models (Bidirectional Encoder Representations from Transformers; Devlin et al., 2018) breaks up the sentence "Open-source LLMs rock." into "[CLS]", "open", "-", "source", "ll", "##ms", "rock", ".", and "[SEP]". This example illustrates that tokens are often words, but not always. They can also be punctuation ("-" and "."), subwords ("ll" and "##ms"), and special tokens ("[CLS]" – shorthand for "classification" – and "[SEP]" – for "separator"). Tokenizers can differ between models and include lower- and upper-case tokens.

**Fig. 2:**
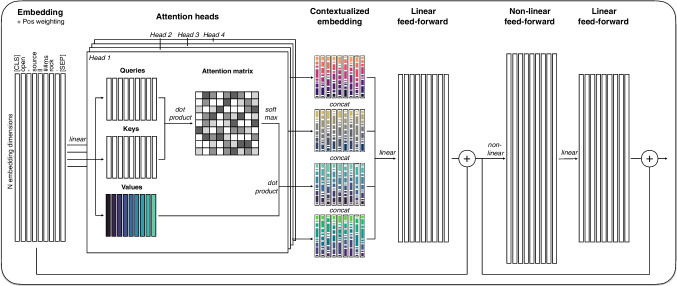
Transformer attention block. The diagram illustrates how the input embeddings are passed to multiple attention heads, each performing a series of operations to generate queries, keys, and values, and leading ultimately to contextualized embeddings

There are several principles behind tokenization. First, including punctuation helps the LLM represent the logical structure of sentences and produce text that contains sentence clauses or multiple sentences. Second, the use of subwords significantly reduces the number of tokens the LLM must learn by assigning stand-alone tokens only to frequent words and constructing all other words using subword tokens. Note that subword tokens that do not start a word begin with "##" to signify that they follow the previous token without a space. Whether a word is assigned a stand-alone token or decomposed into subwords depends on the specific algorithm and the text corpus used by the algorithm to identify the most effective set of tokens. Which words the tokenizer assigns stand-alone tokens to has been found to have important implications for what an LLM learns (Ali et al., 2023). Third, placing special tokens at the beginning ("[CLS]") and end ("[SEP]") of texts enables the LLM to predict the first word and the end of a text and to learn numerical representations that can stand in as a representation of the entire text.

Before we continue, we should note that consistent with much of the literature on large language models (e.g., Devlin et al., 2018), our use of the term "token" does not follow the distinction between types and tokens in philosophy and linguistics, where tokens refer to specific instances or occurrences of types (Wetzel, 2018). Thus, here and in the literature on LLMs, the term "token" refers to both types and tokens.

### Input embeddings

After tokenization, each token is assigned a numeric vector. Prior to training, this simply consists of random numbers. In the case of certain BERT and GPT-2 models (i.e., DistilBERT, BERT-Base, and GPT-2 Small), these vectors consist of 768 numbers (dimensions). The embedding vectors mark the starting point of the neural network and are learned during training, where their values are adjusted iteratively to assist the model in achieving its objective. After training, the input embeddings reflect, in an abstract fashion, a context-general meaning of each token relative to the other tokens.

In contrast to other approaches to semantic representation (Li, 2022; Siew et al., 2019), such as those treating words or tokens as nodes in a network, vector-based embeddings have two key advantages. First, they permit LLMs to be more efficient: The number of embedding dimensions is typically at least an order of magnitude smaller than the number of tokens, resulting in substantially fewer parameters being needed to represent the relationships between tokens. For instance, the WordPiece tokenizer used by BERT encompasses about 30,000 tokens, which is roughly 40 times more than the number of embedding dimensions (i.e., 768). Second, they assist the model in performing generalization: The embedding vectors encode the relationships between tokens to one another, such that the model shows similar behavior for similar tokens.

Input embeddings do not reflect the location of tokens within a given input or context. To capture order, most LLMs, such as GPT-2 and successors, include another component called positional encoding. This is a second token embedding that represents the relative position of tokens using a combination of sine and cosine functions. The positional encoding is typically added to the input embedding to form embeddings that reflects both the context-general meaning of a token and its position in the input to the model.

### Attention blocks

The attention block is the central building block of transformer models and is what distinguishes them from other neural network-based language model architectures, such as *Word2Vec* (e.g., Mikolov et al., 2013) and recurrent neural network-based language models (Graves, 2012). The purpose of the attention block is to produce embeddings that represent the in-context meaning of tokens. For example, consider again the sentence "Open-source LLMs rock." After input embedding and positional encoding, each token in the sentence is represented using a context-general embedding vector. These context-general embeddings reflect the meaning of tokens broadly, not considering the specific context in which they occur. However, the meaning of tokens can vary considerably across contexts: consider, for example, the polysemous "rock." The transformer architecture uses the attention mechanism to capture these context-specific meanings.

The components of the attention block are illustrated in Fig. 2. It begins with the token embeddings, which, in the case of the first attention block, are the sum of the input embeddings and the positional encoding, normalized to have a zero mean and unit variance. Entering the attention mechanism, these embeddings are transformed by a linear layer into three new, lower-dimensional embeddings called *queries*, *keys*, and *values*. This transformation can be likened to a method – principal component analysis – known to behavioral researchers in that it produces output variables that are lower dimensional linear combinations of the input variables. The names of these three smaller embeddings suggest that they can be likened to a retrieval, where a query is compared to keys to identify a value matching the query. Although this analogy should not be taken too literally, it does reflect how the *queries*, *keys*, and *values* collaborate to produce contextualized embeddings for each token. Specifically, the *queries* and *keys* combine to determine how to recombine the *values* to build contextualized embeddings.

Computationally, the attention mechanism works as follows. First, the dot product of each pair of *queries* and *keys* is computed, forming a square matrix of *attention scores*. The attention scores are then normalized by $\sqrt{d_k}$, to account for the dimensionality *d* of the *keys* *k*. These normalized attention scores are next fed row-wise into the softmax function $e^{x_{i}}/\sum _j e^{x_{j}}$, where each $x_i$ is a scalar attention score on *key* *i*, to produce *attention weights* $w_{ij}$. Finally, the attention weights of each row, which now add to 1, are used to produce a weighted sum $\sum _j w_{ij} * v_j$ of the *values* *v* of all tokens. These weighted sums are the new contextualized embeddings for each token. This attention mechanism can also be represented using the following matrix notation

$$ \text {Attention}(Q, K, V) = \text {softmax}\left( \frac{QK^T}{\sqrt{d_k}}\right) V $$

where *Q*, *K*, and *V* are matrices respectively containing the *queries*, *keys*, and *values*, and *T* denotes the matrix transpose. This process of generating contextualized embeddings by mixing *values* as prescribed by the *queries* and *keys* does not per se produce useful contextualized embeddings. This is only achieved in training, which allows the model to figure out how to construct *queries*, *keys*, and *values* such that the contextualized embedding helps it achieve the model objective, such as predicting the next unit in a sequence (see Section *Model output and training*). In other words, the model uses the attention mechanism to learn how context can help it solve a prediction problem.

The next step in the attention block is to gather the contextualized embeddings from the multiple attention mechanisms that are executed in parallel across several attention heads. Running multiple attention mechanisms in parallel permits the model to produce different contextualized embeddings – based on different *queries*, *keys*, and *values* – that may focus on different kinds of relations between tokens. However, it should be noted that these relations are typically not human-interpretable (e.g., Vig, 2019; Vig & Belinkov, 2019). Figure 2 shows , for illustrative purposes, four attention heads; however, current models usually have significantly more. The contextualized embeddings from the four attention heads are concatenated such that the final embedding for each token is a combination of the embeddings produced by the different heads. This final contextual embedding has the same dimensionality as the input embeddings (e.g., 768).

The attention block further processes the contextualized embeddings in several steps. First, they are passed through a linear layer. They are then added to the initial embeddings that entered the attention heads. This addition is called a skip connection and is thought to help stabilize the model during training. After the skip connection, the embeddings are passed through a larger layer with a nonlinear activation function that plays two important roles. First, through its nonlinearity, it provides considerable flexibility in processing and recombining the contextualized token embeddings. Second, through its larger number of nodes, it provides a number of weights (parameters) that enhance the network’s memory capacity. In the case of BERT models, this nonlinear layer is four times larger than the token embeddings, implying an upscaling to 3072 dimensions and over two million weights connecting the two layers. After the large, nonlinear layer, the token embeddings are scaled back down to the standard embedding size using a linear layer, so as to match the required input size for the next model block, and passed through another skip connection prior to the nonlinear layer to provide stability.

Ultimately, the attention block produces embeddings for each token that are the same size as the initial embeddings but are now contextualized such that each token’s embedding is a complex recombination with the other tokens’ embeddings. Following the first attention block, most transformer models add additional attention blocks that take the contextualized embeddings from the previous block as input. As a result, transformer models produce several layers of abstraction where the final contextualized embeddings are combinations of recombinations (for more technical introductions to the attention mechanism, see Prince, 2023; Tunstall et al., 2022; Sanderson, 2019).

**Fig. 3:**
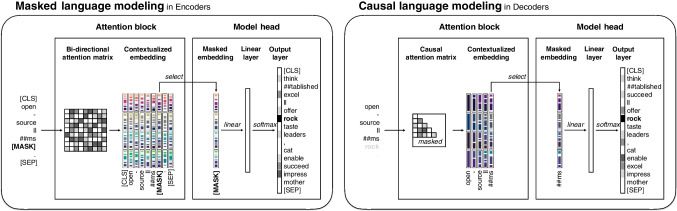
Overview of pre-training mode and transformer family. The figure illustrates two pre-training modes (masked language modeling, causal language modeling) and associated architecture family (encoder, decoder)

### Model heads and training

The final component of the model is the model head. This produces the model output, which can be adjusted to numerous tasks. During *pre-training* – that is, the initial phase of training a language model on a large corpus by learning linguistic patterns and relationships within the data – the model head usually performs token classification. This means predicting one or more of the tokens in the model’s vocabulary based on the model input. There are two dominant approaches to pre-training LLMs through token classification: *masked language modeling* and *causal language modeling*. Of course, given the availability of high-quality, pre-trained LLMs, we believe most behavioral scientists will have little reason to train their own LLM from scratch. Furthermore, the computational resources and technical expertise required to do so can be prohibitive. However, discussing these two modes of training will both help in illustrating the role of a model head (see Fig. 3) as well as provide some necessary background for making informed decisions about which kind of pre-trained model to use for the task at hand (see Section “Choosing between models”).

In *masked language modeling*, a special "[MASK]" token is inserted into the token sequence in place of one or more randomly selected tokens. For instance, in our example sentence, replacing the word "rock" would result in the token sequence "[CLS]", "open", "-", "source", "ll", "##ms", "[MASK]", ".", "[SEP]" as model input. As with any other token, the model will produce an embedding for the "[MASK]" token that reflects its context. This contextual information can thus be leveraged by the model head to predict the masked token. To achieve this, the model head takes the "[MASK]" token’s final contextualized embedding, passes it first through a final hidden layer, which can be linear or nonlinear, and then into the final linear layer with a softmax output that has as many output nodes as there are tokens in the model’s vocabulary. As in the attention mechanism, the softmax function produces values that sum to 1, meaning they can be interpreted as probabilities. The model head uses these probabilities to predict which token is behind the "[MASK]" token. Before pre-training, these predictions will be no better than chance. However, during training, the model will incrementally adjust its parameters ("weights") in a direction that produces higher probabilities for tokens that are behind the "[MASK]" token and lower probabilities for those that are not. In our example sentence, this would mean learning to assign a higher probability for the target, "rock", and other tokens plausibly fitting the context, such as "impress" or "excel", and a lower probability for unfitting tokens, such as "cat", "taste", or ",".

The second and now more popular way to pre-train LLMs is known as *causal language modeling* (or autoregressive modeling). In this mode of training, the model head also performs token classification. However, instead of predicting a masked token from its complete surrounding context, the model is trained to predict the next token in a sequence based only on the tokens that preceded it (i.e., it does not get access to future tokens). To perform this kind of training, causal language models use different tokenizers without a "[MASK]" token. Models trained using causal language modeling also implement a different type of attention mechanism that manually sets all attention to future tokens to zero. This means that each token’s contextualized embedding is composed only of its own *value* vector and the *values* of the tokens preceding it. To predict the next token, the model head selects the contextualized embedding of the last available token of the input (which is used analogously to the "[MASK]" token in masked language modeling). For example, to predict the token "rock" based on preceding tokens, the model head would select the contextualized embedding of the "##ms" token, including information about the preceding tokens and itself, and predict which of all possible tokens likely follow. After training, the model will assign high probabilities to suitable tokens, such as "enable", "offer", or the target token "rock", but not to unsuitable tokens. Importantly, these will not be the same tokens predicted in masked language learning, due to the difference in the information accessible to the model. Specifically, not being able to look into the future, a model trained through the causal language mode would likely assign some probability to the token "," to mark the end of a sentence clause. This is an unlikely prediction for a model trained in masked language mode, which would have access to the period token "." later in that sequence.

Finally, it is important to note that token classification, as employed in masked and causal language modeling, is not the only pre-training objective, nor are masked and causal language modeling the only modes of pre-training. One other pre-training mode for transformer models is *next-sentence prediction*. In this mode, the model head is set up to predict a single numerical value between 0 and 1, reflecting the probability with which two input sentences occurred adjacently in the training data. Next-sentence prediction is used in the pertaining of some BERT-style models, typically in addition to masked language learning. Next-sentence prediction illustrates that the transformer model head can be adjusted to predict different data types. This flexibility is exploited frequently, for instance, in the fine-tuning of LLMs to perform specific tasks based on smaller, task-related datasets (see Section “Fine-tuning”).

### An overview of model types

Since the inception of the transformer architecture (Vaswani et al., 2017), many model variants have emerged that differ in important ways, including the architecture family (i.e., *encoder*, *decoder*, or *encoder–decoder*), model size, stage of training reached by the model, and openness.

Concerning the architecture family, it is helpful to distinguish the *encoder*, *decoder*, and *encoder–decoder* architectures (see Fig. 3). The encoder architecture is characterized by bidirectional attention, pre-training through masked language modeling (and, sometimes, next-sentence prediction), and the use of special tokens such as "[CLS]", "[SEP]", and "[MASK]". The goal of the encoder architecture is to produce accurate contextualized embeddings, including for the special tokens. Prominent examples following the encoder architecture are the models of the BERT family (e.g., DistilBERT (Sanh et al., 2019) or RoBERTa (Liu et al., 2019)), and the instructor models (Su et al., 2022). The decoder architecture, on the other hand, is characterized by causal attention and pre-training through causal language modeling. The goal of the decoder architecture is to generate text via next-token prediction. Prominent examples of the decoder architecture are the GPT (OpenAI, 2023) and LLaMA model families (Touvron et al., 2023). Finally, the encoder–decoder architecture is characterized by a combination of the two, and is the original transformer architecture as proposed in Vaswani et al. (2017). It is trained with next-token prediction, where the input text is first fed to the encoder, the encoder’s last hidden state is then passed as input to the decoder, which then predicts the next token. Prominent examples of the encoder–decoder architecture are the BART (Lewis et al., 2019) and T5 (Raffel et al., 2020) models.

A second key differentiating factor between LLMs is size. Size is often measured in terms of the number of weights in a model, which can vary between a few hundred million (e.g., most BERT models) and several hundred billion (Smith et al., 2022, e.g., Megatron Turing NLG) – or even the trillion weights supposedly reached by OpenAI’s GPT-4 model. Although the number of weights plays a large role in determining a model’s capacity to learn from the training data, how the weights are distributed throughout the various model components also matters (Kaplan et al., 2020). The size of LLMs can also differ in a more functional way, by allowing for different context sizes. The context size is the maximum number of tokens in a sequence that the attention mechanism can evaluate at any given time, and it determines the complexity of connections between tokens that the model can consider. This is important for applications such as few-shot learning (see Section “Applications of LLMs in behavioral science”). From a practical perspective, context size also determines the amount of random-access memory (RAM) needed to run a model. For large decoder models such as LLaMA-2 (70 billion weights), RAM requirements can be as high as 300 gigabytes, resulting in a need for expensive, high-performance graphical processing units (GPUs).

A third differentiating factor is the stage of training reached by the model. First, there are *pre-trained models*, which have been trained on a large corpus of text using masked or causal language modeling. The text corpus typically includes websites, which in turn include blogs, news articles, Wikipedia, social media platforms (e.g., Reddit), and other sources of text (e.g., books, academic articles). Larger pre-trained models are sometimes also called foundation models (Bommasani et al., 2023), emphasizing their purpose as a basis for task-specific training. Second, there are *fine-tuned models*, which are pre-trained models that have been further trained on task-specific data to selectively increase their performance on certain tasks. These can be basic tasks, such as token classification or prediction of numerical variables, or more complex tasks, such as named-entity recognition or question answering.

A fourth differentiating factor that concerns the set of fine-tuned models and has played an especially important role in the growth in popularity of LLMs in recent times is whether the model is a "chat" model. Specific fine-tuning regimes exist to make pre-trained models better suited to high-quality, assistant-style interactions through a chat interface. These include training steps with explicit human input such as *supervised fine-tuning*, *reinforcement learning from human feedback* (Christiano et al., 2017; Touvron et al., 2023), and *direct preference optimization* (Rafailov et al., 2024). For instance, in supervised fine-tuning, human "annotators" generate prompts and appropriate assistant-style responses to those prompts, such that the model may learn via "imitation" to become a good assistant. Reinforcement learning from human feedback is a more complex, multistage procedure in which human annotators indicate their preferences between model outputs according to specific criteria (e.g., safety or helpfulness) to build a "preference dataset" (stage 1) (Touvron et al., 2023). This dataset is then used to train a reward model that learns the annotators’ preferences (stage 2), which in turn provides feedback on the outputs of the LLM in much vaster quantities than would be possible with human annotations alone. This enables the LLM to learn via reinforcement to become a better assistant. Such fine-tuning can be seen as an example of how LLMs can be tailored to specific behavioral applications. A prominent example of a chat model is ChatGPT, but other open-source models exist, including LLaMA-2 Chat (Touvron et al., 2023) or Falcon Chat (TII, 2023).

A fifth and final differentiating factor is openness. LLMs differ in terms of how much information is available about the training data, training method, or architecture (see Bommasani et al., 2023) and how openly available the models themselves are. Some models, such as GPT-4, are only available through remote user interfaces, whereas others are mostly (e.g., LLaMA) or fully (e.g., BERT) open-source. Most open-source models can be accessed and employed via the Hugging Face ecosystem introduced in this tutorial, which has significant advantages over closed-source models in terms of transparency and reproducibility.

## Applications of LLMs in behavioral science

LLMs can be employed in several ways in behavioral science. The most basic but often effective mode is *feature extraction* (e.g., Aka & Bhatia, 2022; Binz & Schulz, 2023a; Hussain et al., 2023; Wulff & Mata, 2023). Feature extraction sends text input through the model and records contextualized embeddings, typically at the final layer. The resulting embedding vectors can then be utilized in many ways. For example, the use cases presented in the next sections demonstrate how feature vectors can be used to predict the similarity between personality constructs, choices in reinforcement learning tasks, or numerical judgments such as risk or health perception ratings. Feature extraction is commonly performed using encoder models with bidirectional attention (Muennighoff et al., 2022), which allows them to better utilize all available information during pre-training. However, when the goal is to predict the next element in a sequence, decoder models can be equally or more effective, also because current decoder models are significantly larger and trained on more data than encoder models.

Another class of applications utilizes the model’s ability to predict outcomes with no or minimal supervision. The use of transformer models without any kind of task-specific training to predict verbal or numerical outcomes is called zero-shot learning. This approach can be used to generate categorical and numerical predictions – for example, to predict the category of a news article or the sentiment of a social media post (e.g., Widmann & Wich, 2023; Pelicon et al., 2020; Gilardi et al., 2023). Zero-shot learning can be performed using all three types of transformer architectures, with some differences in implementation. Few-shot learning is an extension of zero-shot learning where minimal supervision is provided in the model input – for instance, in the form of a handful of input–output pairs. In classifying a social media post’s sentiment, this would imply including pairs of the post’s text and known sentiment in the model input. Few-shot learning can, in principle, be used with any model type; however, it is most commonly employed using modern decoder models, which tend to contain more parameters and show better performance on few-shot tasks (Brown et al., 2020). Off-the-shelf, large-scale decoder models, such as GPT-4, provide good zero-shot and few-shot performance (Törnberg, 2023; Rathje et al., 2023).

However, *fine-tuning* smaller language models on a specific task can result in equally good or even better performance relative to the zero- or few-shot performance of larger models (Wang et al., 2022; Chae & Davidson, 2023; Rosenbusch et al., 2023). Unlike zero- or few-shot learning – both of which leave model parameters unchanged during the "learning" phase – fine-tuning involves explicit updating of model weights with the goal of improving task-specific performance. As a result, fine-tuning is an important strategy for applications in behavioral science (e.g., Demszky et al., 2023).

**Fig. 4:**
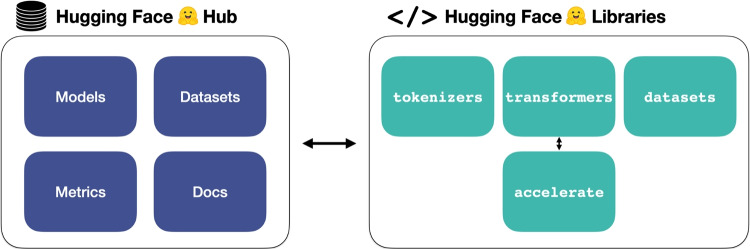
The main components of the Hugging Face ecosystem. The figure is adapted from Tunstall et al. (2022)

The final class of application is *text generation*. This application is specific to encoder-decoder and decoder-only models. Text generation can be used to perform various tasks including summarization, question answering, or simply free text generation. Some examples of the use of free text generation include comparing reasoning abilities in LLMs and humans (Yax et al., 2023; Webb et al., 2023) and simulating the responses of study participants (Argyle et al., 2023). These simulations need not be constrained only to *predicting* human behavior, but could also be used to suggest *explanations* for why people behaved a certain way (Bhatia, 2023).

## Choosing between models

Given the various model types available and their differing applications in behavioral science, one natural question that follows is which model to use for the application at hand. In view of the constantly developing architectural landscape and the important role played by task-specific properties, it is difficult to predict model success. Nevertheless, some heuristics tend to hold.

First, larger models tend to perform better than smaller ones (Kaplan et al., 2020). On the other hand, they also demand more computational resources. Modern large language models typically exceed the capacity of personal computers, requiring access to either high-performance clusters or cloud computing services. These requirements can be alleviated with quantization (e.g., Ma et al., 2024; Frantar et al., 2022), reducing a model’s numerical precision, or knowledge distillation (Hinton et al., 2015), transferring a model’s representations into a smaller model. Although these approaches usually come with some loss in performance, a quantized version of a larger model will, for instance, often outperform its smaller, full-precision equivalents. Ultimately, choices concerning model size must factor in these kinds of computational resource considerations.

Second, decoder models are more suited than encoder models to tasks where tokens must be predicted in sequence – that is, in the order that the tokens would be written (see, e.g., Section *Extracting token probability and perplexity*). This is due to their causal language modeling pre-training objective, which prevents them from "cheating" by having access to future tokens when predicting the present token. On the flip side, when representations of the context (before and) after a given token of interest are desired (see, e.g., sections *Relating personality measures* and *Predicting health perception*), encoder models typically outperform similarly sized decoder models. This is often the case for feature extraction tasks unless the goal is to generate predictions that are more sequential in nature (see, e.g., Section “Predicting repeated choice”), in which case a decoder model may be better suited.

Third, chat models that have been fine-tuned with assistant-oriented regimes, such as reinforcement learning from human feedback, are often better at producing coherent human-like output. This makes them more useful for a variety of tasks, such as text labeling, information retrieval, and text generation. The latter includes applications such as generating study materials or simulating human participants in behavioral experiments (see Section “Text generation”).

In addition to these heuristics, several empirical benchmarks exist that can help with selecting the right model for a task. There are separate benchmarks for various tasks, covering both feature extraction (e.g., huggingface.co/spaces/mteb/leaderboard) and text output (e.g., huggingface.co/spaces/lmsys/chatbot-arena-leaderboard). An overview of benchmark results for open models accessible through Hugging Face can be accessed at huggingface.co/spaces/HuggingFaceH4/open_llm_leaderboard. Generally, it is useful to select the model whose benchmark performance is high for tasks similar to the task at hand. Of course, benchmark performance may fail to generalize.

## The Hugging Face ecosystem

The Hugging Face ecosystem has two main components: an online hub ("the Hub", huggingface.co/docs/hub) and a set of Python libraries (as illustrated in Fig. 4, huggingface.co/docs). Hosting over 300,000 models and 60,000 datasets, the Hub constitutes an impressive community-driven effort to democratize language modeling, as well as other types of modeling, such as computer vision. Furthermore, thanks to Hugging Face’s Python libraries, many of the steps traditionally required to implement LLMs have become considerably more accessible, often requiring just a few lines of code each. These steps typically include processing the data, initializing the model, and applying the model to a specific task. This section introduces some of the more crucial components of libraries, such as |datasets|, |tokenizers|, |transformers|, and |accelerate|, and outlines how these components can help with these tasks.

The first step in almost any language modeling pipeline is data processing. This usually involves data loading, cleaning, and reshaping. Because natural language processing (NLP) datasets can sometimes be in the order of tens or even hundreds of gigabytes, they cannot always be loaded into RAM or stored on the hard drive. |datasets| addresses this issue by enabling users to convert their data to Apache Arrow format, allowing it to be flexibly and efficiently read from the hard drive or streamed online, thus preventing RAM or hard drive storage overload.

**Table 1:** Three example items from IPIP-NEO-300 five-factor personality inventory Kajonius & Johnson (2019)

Text	Construct	Factor
Go straight for the goal.	Achievement-Striving	Conscientiousness
Plunge into tasks with all my heart.	Achievement-Striving	Conscientiousness
Remain calm under pressure.	Vulnerability	Neuroticism

Once the dataset has been loaded in |datasets. Dataset| format, it must be tokenized before it can be fed into a model. It is essential that the method of tokenization is matched to the pre-trained model. Hugging Face’s |tokenizers| and higher-level API alternatives from the |transformers| library make it easy to initialize the appropriate tokenizer using the tokenizer’s .from_pretrained() method. This can be done by passing the model *checkpoint* – a unique character string that identifies the model on the Hugging Face Hub (see huggingface.co/models).

Hugging Face models can be loaded in a single line using the transformers.AutoModel.from_pretrained() method, and placed on the graphics processing unit (GPU) if a compatible GPU is available. This can speed up model inference and fine-tuning to such an extent that it may make an otherwise infeasible task feasible. Training and inference can be further optimized by distributing across multiple GPUs with |accelerate|. In the examples that follow, |accelerate| works in the background of the |transformers| library via arguments such as device_map="auto" to automatically optimize the distribution of resources across processing units to allow easy upscaling to larger models.

It is important to note that the Hugging Face ecosystem is dependent on deep learning libraries such as PyTorch (pytorch.org) or TensorFlow (tensorflow.org), and interacts with popular Python libraries such as NumPy (numpy.org) and Pandas (pandas.pydata.org). Furthermore, it interfaces with several other Python libraries, including SentenceTransformers (sbert.net/docs/quickstart.html), offering a high-level API to obtain embeddings from over 500 models hosted on the Hugging Face Hub in addition to providing its own pre-trained models.

In what follows, we demonstrate how the Hugging Face ecosystem can be used for three types of applications, namely, feature extraction, fine-tuning, and text generation, by presenting several use cases in behavioral research with (accompanying code). Comprehensive and richly commented code is available in notebook format at github.com/Zak-Hussain/LLM4BeSci.git, a GitHub repository with instructions for running the code online in a Google Colab environment. The repository also provides a means of keeping the code base for this tutorial up to date. Keep in mind that the Hugging Face ecosystem is in active development, making it likely that specific aspects of the code presented in this paper will be deprecated by the time of reading. We plan to regularly update the GitHub repository and respond to update requests, which can be submitted as GitHub issues at github.com/Zak-Hussain/LLM4BeSci/issues/new. For further information on Hugging Face, we suggest the Hugging Face textbook by Tunstall et al. (2022).

## Feature extraction

### Relating personality measures

Feature extraction from LLMs is already being leveraged in diverse ways to assist research in personality psychology (e.g., Abdurahman et al., 2023; Cutler & Condon, 2023; Wulff & Mata, 2023). In this example, we show how LLMs can be used to predict the relationship between existing personality measures (i.e., personality items and constructs). Specifically, we walk the reader through an analysis pipeline emulating the work of Wulff & Mata (2023) that used feature extraction to generate item and construct embeddings – representations of psychometric items and constructs in a vector space obtained from LLMs – and then applied these vectors in downstream tasks, such as similarity comparison and clustering, to both validate different models and tackle the lack of conceptual clarity in personality psychology (Wulff & Mata, 2023). See github.com/Zak-Hussain/LLM4BeSci.git to run this example in a Google Colab environment.

The example begins by loading the relevant data, in this case, data concerning the IPIP-NEO-300 five-factor personality inventory (Goldberg et al., 2006), into a pandas.DataFrame, verb|neo_items (see Table 1). The goal in this example will be to obtain item embeddings – representations of personality items and constructs – using feature extraction, so that these embeddings can be used to estimate the similarity between items and constructs. The similarity between items and constructs can ultimately be used to uncover the structure of psychological constructs and to inform the labeling of these measures (Wulff & Mata, 2023). The data set used in the example has three columns: |’item’| (the personality item description), |’construct’| (the personality construct to which the item belongs), and |’factor’| (the Big Five personality factor to which the construct belongs). Once the data has been loaded (and any necessary cleaning and reshaping performed), they are converted into a |datasets.Dataset| object for efficient storage using the from_pandas() method.

The text input is now ready for tokenization. As mentioned earlier (see Section “Tokenizers”), the tokenizer must be consistent with the model to be used downstream. As such, a model checkpoint (model_ckpt) must first be defined. In our example, we use a lightweight version of BERT (|’distilbert-base-uncased’|) to ensure ease of storing and running the model on most hardware setups. However, it could easily be replaced by a larger model from the Hugging Face Hub, hardware limitations permitting. With the model_ckpt specified, the model tokenizer can be loaded with AutoTokenizer.from_pretrained(model_ckpt).

Tokenization is performed efficiently across groups of inputs, called batches, by mapping |dat| with a user-defined batch_tokenizer function. This takes two important arguments: |padding| and |truncation|. |padding| is used to fill up sequences with zeros to match the length of the longest sequence in the batch, thus ensuring that all sequences in the batch have the same length. This is essential for training and inference with deep learning models, which operate on fixed-size input tensors. Tensors are a generalization of vectors, including vectors, matrices, and higher order arrays. |truncation| is the process of cutting off the end of a sequence to ensure that it fits within the model’s maximum context size. In the case of BERT models, this is 512 tokens. It is worth noting that alternative strategies exist if the to-be-dropped part of the sequence is thought to contain useful information, including splitting up the sequence into digestible batches and averaging the embeddings across batches.

As output, the batch_tokenizer returns a Python dictionary with two keys: ’input_ids’ and ’attention_mask’. ’input_ids’ maps to a list of integers uniquely representing each token in the sequence. ’attention_mask, which is not to be confused with the learned attention weights, maps to a list of ones and zeros, where the ones stand in place of each token and the zeros pad the sequence to match the longest in the batch due to |padding|. For instance, the personality item |’Go straight for the goal.’| tokenizes to:

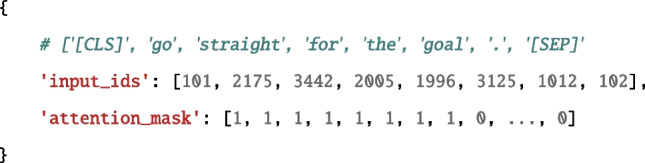



with the ’input_ids’ referring to the tokens "[CLS]", "go", "straight", "for", "the", "goal", ".", and "[SEP]". The final pre-processing step involves converting the data to the PyTorch (|torch|) format such that they can be passed to the model.

The data are now ready to be fed into the model. Model architecture and pre-trained weights are loaded in a single line with AutoModel.from_pretrained(model_ckpt). The code next detects whether a Compute Unified Device Architecture (CUDA)-compatible or Apple Metal Performance Shader (MPS)-compatible GPU is available using PyTorch and, if so, sets the device to the GPU. Otherwise, the CPU is used. The model is then moved to the device with the |to()| method.

Data can be more efficiently fed into the model in batches and this is done automatically using the |Dataset.map()| method. To extract the features (i.e., the numerical vector representations of the inputs), the researcher must define a function extract_features(). It takes batches of |dat| as input and selects the columns containing the model inputs by checking whether the column name is referenced in a list accessed via tokenizer.model_input_names. In this case, the list contains two model inputs: ’input_ids’ and ’attention_mask’. These are then input to the model with gradient calculations disabled by torch.no_grad(). This is done for efficiency reasons: By default, |torch| models build a computational graph in the background in order to perform gradient descent. Because feature extraction only performs a forward pass through the model (i.e., there is no weight updating), no computational graph is required.

Finally, the extract_features() function extracts the activations of the last layer of the model via model(**inputs).last_hidden_state. This returns a tensor of shape batch_size, n_tokens, hidden_dim – in this case |8, 16, 768|, respectively – due to being passed through |dat.map()| with batch_size=8, padding=True , and the number of embedding dimensions in the model being 768. It is worth mentioning that because padding=True pads to the length of the longest sequence in the batch, n_tokens will not always equal 16. From this tensor, the first token features at position |0| are selected, moved back onto the CPU, and converted to a NumPy array per the requirement of |Dataset.map()|. The first token is the special "[CLS]" token, inserted at the beginning of each input. It is known to produce a holistic representation of the content of the entire input and is therefore a common target in feature extraction (Tunstall et al., 2022). However, it should be noted that other feature extraction strategies exist than those focusing on the "[CLS]" token, such as taking the average of all output representations (known as "mean-pooling", see, e.g., Reimers & Gurevych, 2019).

The function is then applied using the |dat.map()| method, which runs the items in batches of eight through the function. Depending on the researcher’s RAM constraints, the batch_size could be increased. Finally, the features are converted into a |pandas.DataFrame| for later downstream use.

In our example, the application of item embeddings involves similarity comparison between personality items. The similarity between the items is evaluated by passing dat[’hidden_state’], containing the features extracted for each item, to |sklearn|’s cosine_similarity() function. This function computes the cosine similarity, a measure commonly used to evaluate the similarity between embedding vectors, for each pair of items and returns a square NumPy array of pairwise item similarities.

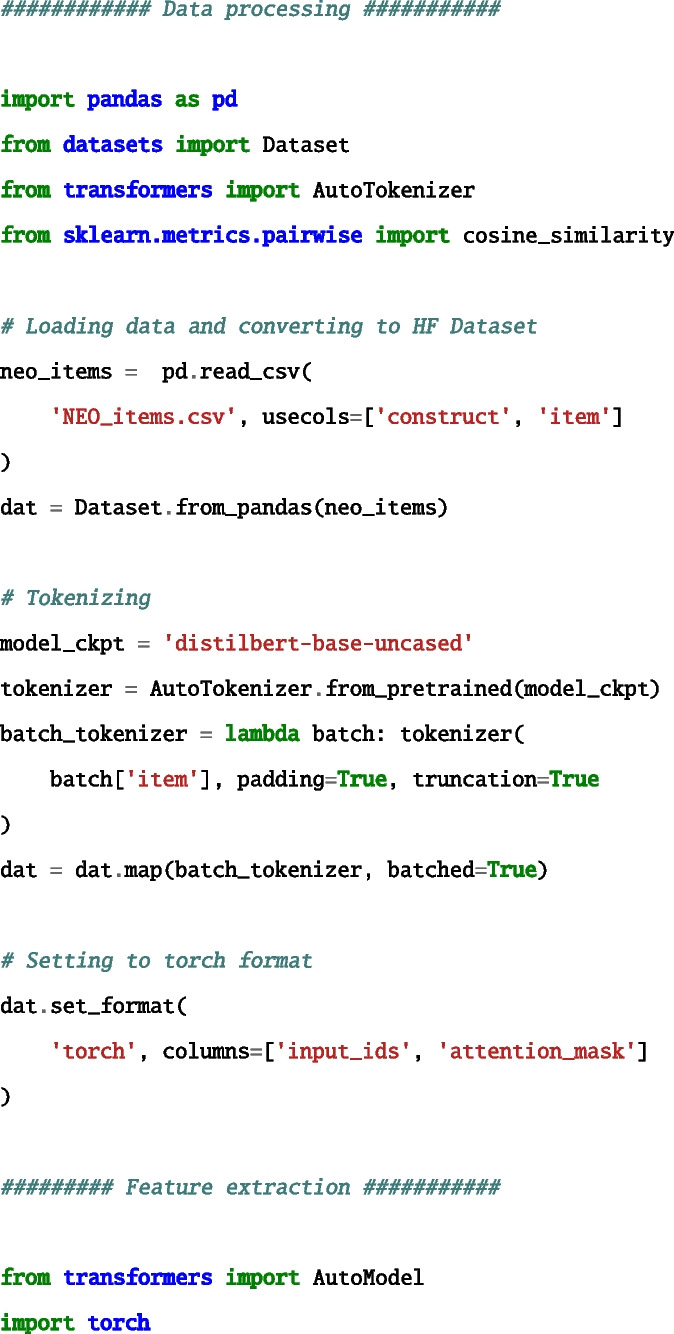

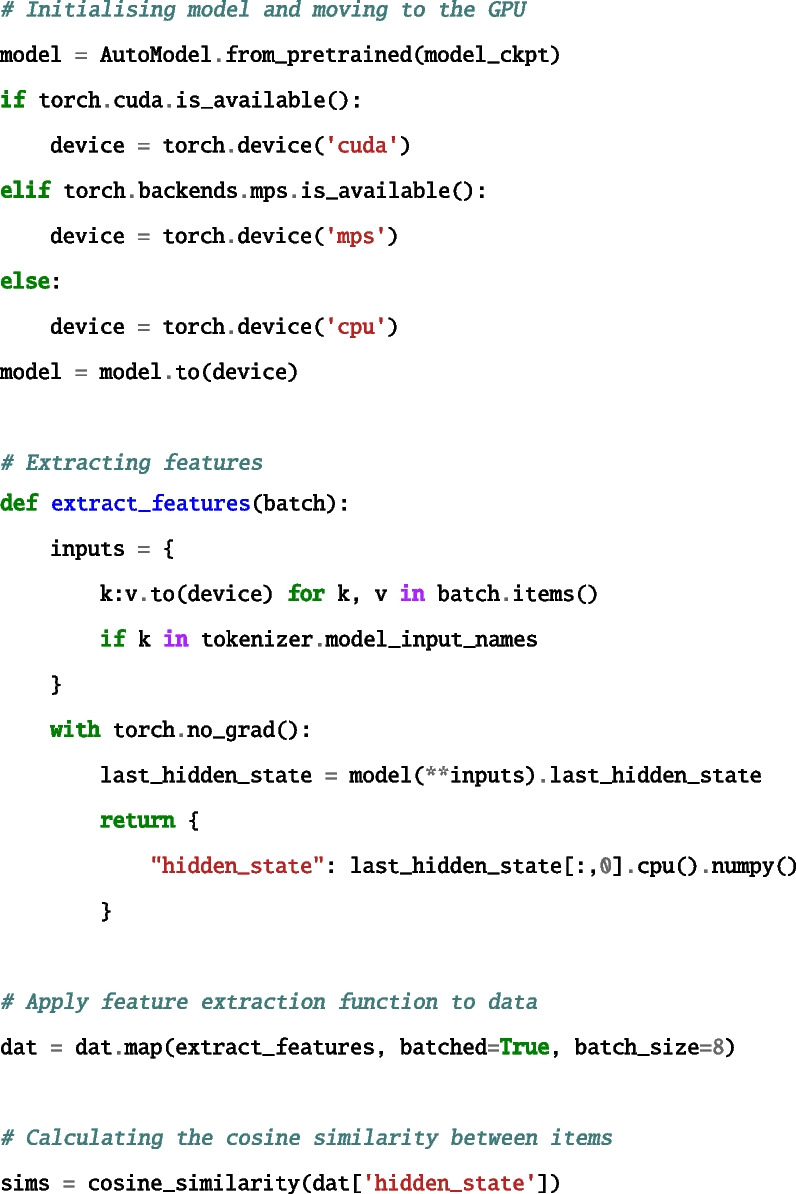





Before we discuss the actual use of the cosine similarities obtained, it should be noted that there are higher-level API alternatives to feature extraction, such as the SentenceTransformers library (sbert.net/docs/quickstart.html) and the Hugging Face |transfomers.pipeline| abstraction. There are, however, two reasons for introducing feature extraction as we do above. First, even if the researcher opts for the higher-level API in their own work, the lower-level code can give them a better understanding of what is happening in the background. For instance, it highlights the importance of tokenization, allows researchers to easily inspect which inputs get fed into the model (’input_ids’ and ’attention_mask’), and indicates which features get extracted (the last hidden state). This background understanding can be crucial for debugging code and knowing how to appropriately adjust it to the research context. Second, and almost by definition, the lower-level API has the advantage of greater customizability. That being said, due to their simplicity, higher-level APIs will often be the best option for researchers wishing to implement their own feature extractor. For the sake of brevity and consistency, we demonstrate how this can be done with the higher-level |transformers.pipeline| API, and refer readers to the SentenceTransformers library for an alternative approach.

The pipeline object, |pipeline|, achieves the same results as the example above but with considerably less code. |pipeline| takes |’feature-extraction’| as a positional argument upon initialisation. In the same line, the desired model and corresponding tokenizer can be loaded by specifying model=model_ckpt and tokenizer=model_ckpt as arguments. Rather than moving the data and model onto the GPU with the |to()| method, this is all done in the background by setting |device=device|. PyTorch is specified as the chosen deep-learning framework for running the model with |framework=’pt’|. Finally, feature extraction is run by passing the personality items as a list of strings to the now initialized feature_extractor, with tokenization options such as padding and truncation provided as a dictionary argument via tokenize_kwargs. The feature extraction returns a list of tensors with each tensor corresponding to a sample (i.e., personality item), and with the "[CLS]" features accessible at index |[0, 0]|.

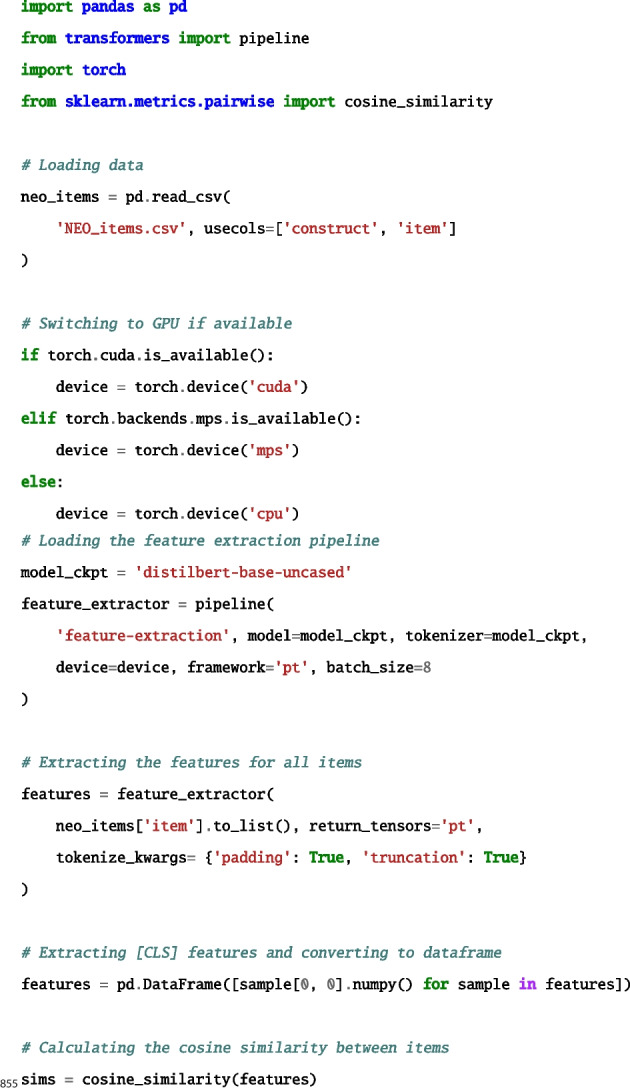



**Fig. 5:**
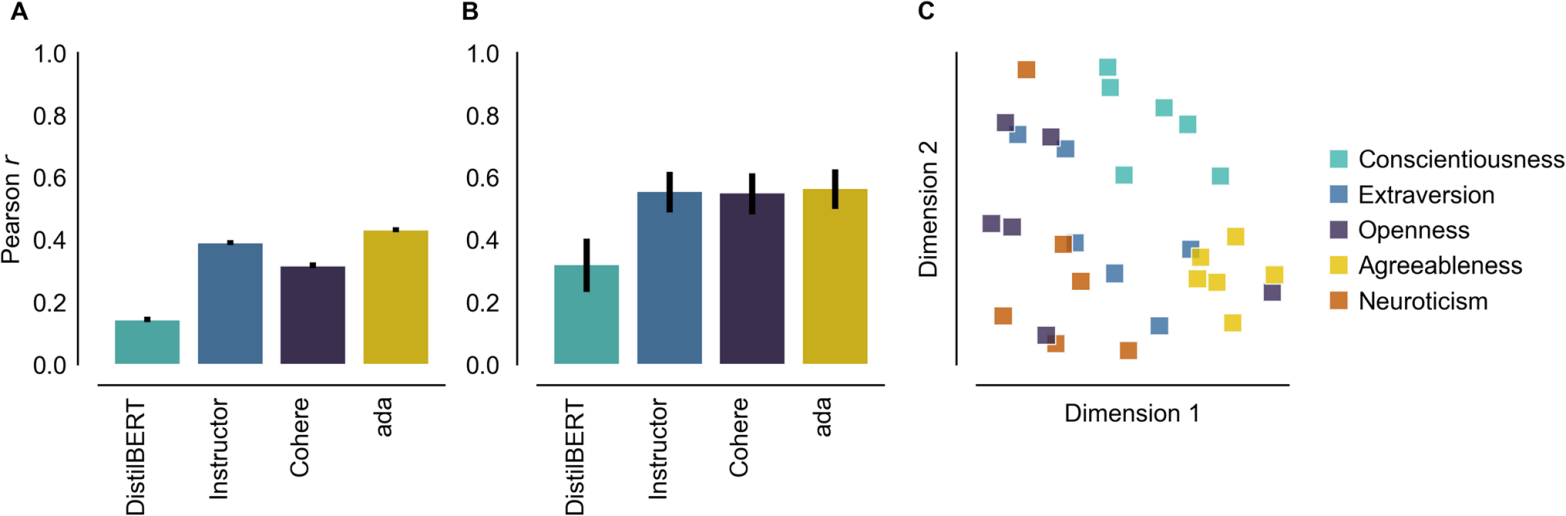
Personality psychology application. (**A**) Correlations between predicted versus observed item similarities and (**B**) predicted versus observed construct similarities based on embeddings from DistilBERT, Instructor (instructor-xl), Cohere (Cohere-embed-english-v3.0), and ada (text-embedding-ada-002) models. **C** Pairwise controlled manifold approximation projection Wang et al. (2021) of Instructor-XL construct-level features. *Colors* reflect the Big Five personality factor to which the construct belongs. *Error bars* represent 95% confidence intervals

Following Wulff & Mata (2023), the cosine similarities between features can be compared with observed correlations between participant responses at both the item and the construct level. With |’distilbert-base-uncased’|, a relatively simple baseline model, the semantic content of the personality items – as captured by the extracted features – is correlated with the absolute observed similarities with a Pearson $r=.14$. By aggregating item features and item correlations to the construct level, this correlation increases to $r=.32$ (see Fig. 5, panels A and B). As further shown in Wulff & Mata (2023), these correlations are higher for more recent, larger models. For instance, |hkunlp/instructor-xl| is a considerably larger alternative to DistilBERT that contains 1.2 billion as opposed to 67 million parameters. At the time of writing, it is at the top end of the Massive Text Embedding Benchmark (MTEB) Leaderboard (huggingface.co/spaces/mteb/leaderboard), and is also openly available through the Hugging Face Hub. As such, it presents a promising alternative to DistilBERT. Indeed, it achieves considerably higher item-wise ($r=.39$) and construct-wise ($r=.56$) correlations, which are comparable to those produced by top-of-the-range paid-API-access embedding models from Cohere (*Cohere*, Cohere-embed-english-v3.0) and OpenAI (*ada*, text-embedding-ada-002). Finally, as illustrated in Fig. 5C, plotting a two-dimensional projection of the similarities between constructs reveals that the placement of constructs largely recovers the Big Five personality factors to which the constructs are assigned. Overall, this example highlights that item embeddings generated through feature extraction can accurately capture the empirical correlations between personality items and constructs and the overall structure of human personality, although performance differs across models.

More generally, we should point out that LLMs are not only capable of reproducing known facts about personality psychology. Their ability to capture the conceptual relationships between items, constructs, and their labels has been exploited by Wulff & Mata (2023) to produce a mapping of personality constructs and associated labels that increases parsimony and reduces *jingle–jangle fallacies*; that is, the proliferation of measures that have been given similar labels, yet capture different constructs (jingle fallacies), as well as measures that have received different labels, yet capture the same construct (jangle fallacies). Consequently, feature extraction can be a powerful tool in contributing to conceptual clarity in this field.

### Predicting health perception

In this section, we move into the domain of prediction, with the goal of showing how behavioral researchers can use LLMs to predict human judgments and decisions using the now-familiar feature extraction approach coupled with regression modeling (or other predictive modeling approaches). We use the example of predicting health perceptions following the approach of Aka & Bhatia (2022). We believe that predictive applications of LLMs such as this present a promising means of both tracking real-world perceptions and behaviors (e.g., Hussain et al., 2023), as well as enabling (in-silico) testing of potential interventions for improving communication between, for instance, health experts or policymakers and the general public Aka & Bhatia (2022). See github.com/Zak-Hussain/LLM4BeSci.git to run this example in a Google Colab environment.

The dataset used here has two columns: |’text’| and |’labels’| (Table 2; Aka & Bhatia, 2022). |’text’| is composed of short descriptions of various health states extracted from the U.K. National Health Service’s web page. |’labels’| contains average numerical ratings of the severity of these health states from 782 participants, with higher ratings indicating less severe states. The goal is to build a model that predicts people’s perception of the severity of the health states presented.

As demonstrated in the last section, the hidden state representation of each health description in the |’text’| column can be extracted using |’distilbert-base-uncased’|. These features can then be converted to a pandas.DataFrame, and used as predictors in a regression model to predict the health ratings. So as not to repeat code, this section begins with these features already extracted.

Model performance is evaluated out-of-sample. In the simplest case, this involves splitting the data into a training and a test set using |sklearn|’s train_test_split, with 80% used for training and 20% for testing (as determined by test_size=.2). randome_state=42 is used for reproducibility.

It is important to remember that the extracted features are high-dimensional. In this case, there are 768 predictors, as determined by the number of hidden dimensions in |’distilbert-base-uncased’|. With only 621 samples in the training set, there are more predictors than samples. In such a case, an ordinary least squares regression solution cannot be identified. To address this issue, and more general issues associated with high-dimensional data such as over-fitting and multicollinearity, researchers commonly employ regularization. In this case, |sklearn|’s |RidgeCV| is used, where the regularization penalty (|alpha|) is automatically tuned using cross-validation within the training set.

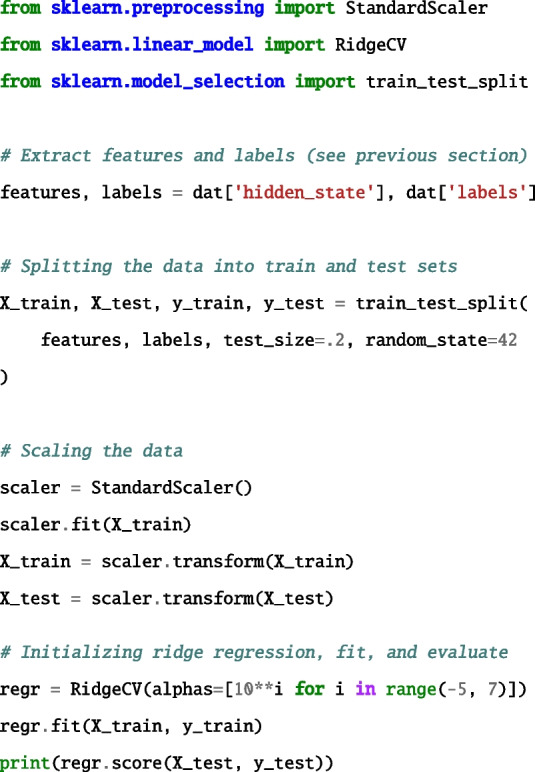



Once the regression model has been initialized using |RidgeCV()| and assigned as |regr|, it is then fitted to the standardized training data with |regr.fit()|. Standardization is commonly performed in regularized regression, such as ridge regression, to ensure that all predictors are given equal weight in the regularization penalty. Finally, model performance is evaluated with |regr.score()|.

**Fig. 6:**
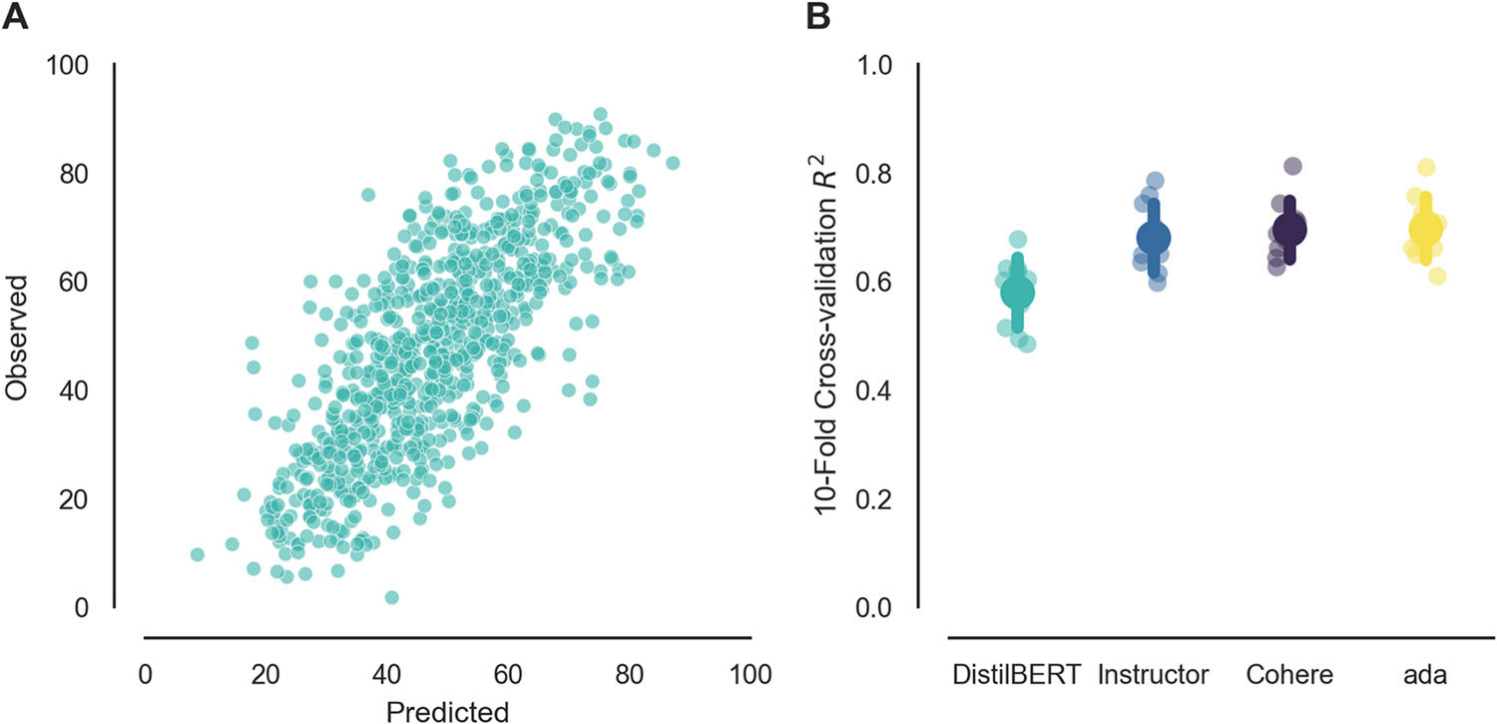
Predicting health perception. **A** Predicted versus observed health ratings using DistilBERT. **B** Comparing the performance of DistilBERT, Instructor (instructor-xl), Cohere (Cohere-embed-english-v3.0), and ada (text-embedding-ada-002), with tenfold cross-validation using ridge regression. *Error bars* reflect $\pm 1$ SD

Figure 6A shows a high alignment of predicted and observed health state ratings, implying that much of the variance in health state ratings can be explained by the DistilBERT features.

**Table 2:** Three examples from Aka & Bhatia (2022)’s health dataset containing short health descriptions from the U.K. National Health Service’s web page and average participant ratings of the severity of these health states

Text	Label
Broken leg. A broken leg (leg fracture)...	49.33
Bulimia. Bulimia is an eating disorder...	34.18
Hyperacusis. Hyperacusis is when...	53.82

For the purposes of keeping the tutorial code simple, out-of-sample performance was measured using a single train-test split with a single model; ideally, performance would be evaluated across multiple splits to ensure the robustness of the results. Using ridge regression with automatic (nested) tuning of the regularization penalty hyper-parameter (Varma & Simon, 2006), feature extraction with DistilBERT on average explains over half of the variance in health perceptions ($R^2=.58$). This performance, although considerable, is inferior to that of larger models. As Fig. 6B shows, the open-source *Instructor* model (instructor-xl), as well as the proprietary *Cohere* (Cohere-embed-english-v3.0) and *ada* (text-embedding-ada-002) models benefit from their larger size, all showing a relatively similar increase in performance relative to DistilBERT, with the best-performing model achieving $R^2=.70$. These results could potentially be improved further by using different prediction algorithms, including nonlinear algorithms, or more fine-grained hyper-parameter tuning.

Overall, the analysis shows that LLMs implementing a simple feature extraction coupled with a regression-based procedure can achieve impressive performance when tasked with predicting human judgments.

### Predicting repeated choice

The previous examples have demonstrated that LLM feature extraction can be used to predict aspects of human psychology and judgments. But what about more complex human behavior? This section demonstrates that the feature extraction pipeline can also be applied to decisions in a repeated choice paradigm that involves sequential cognitive reasoning. Moreover, it shows that only minor code changes are needed to employ some of the largest and most advanced LLMs currently available for these purposes. See github.com/Zak-Hussain/LLM4BeSci.git to run this example in a Google Colab environment.

The experimental data in this section come from a paradigm known as the horizon task (Wilson et al., 2014). In this task, participants repeatedly choose between two options. Upon selecting an option they receive a probabilistic reward. Each game starts with four observational trials, in which the examples are predetermined by a computer program, followed by either one or six choices. Participants are instructed to accumulate as much reward as possible over the experiment. The data considered here are combined from two previous studies (Wilson et al., 2014; Feng et al., 2021) in which 60 participants each played 320 games, making a total of 67,200 choices.

**Fig. 7:**
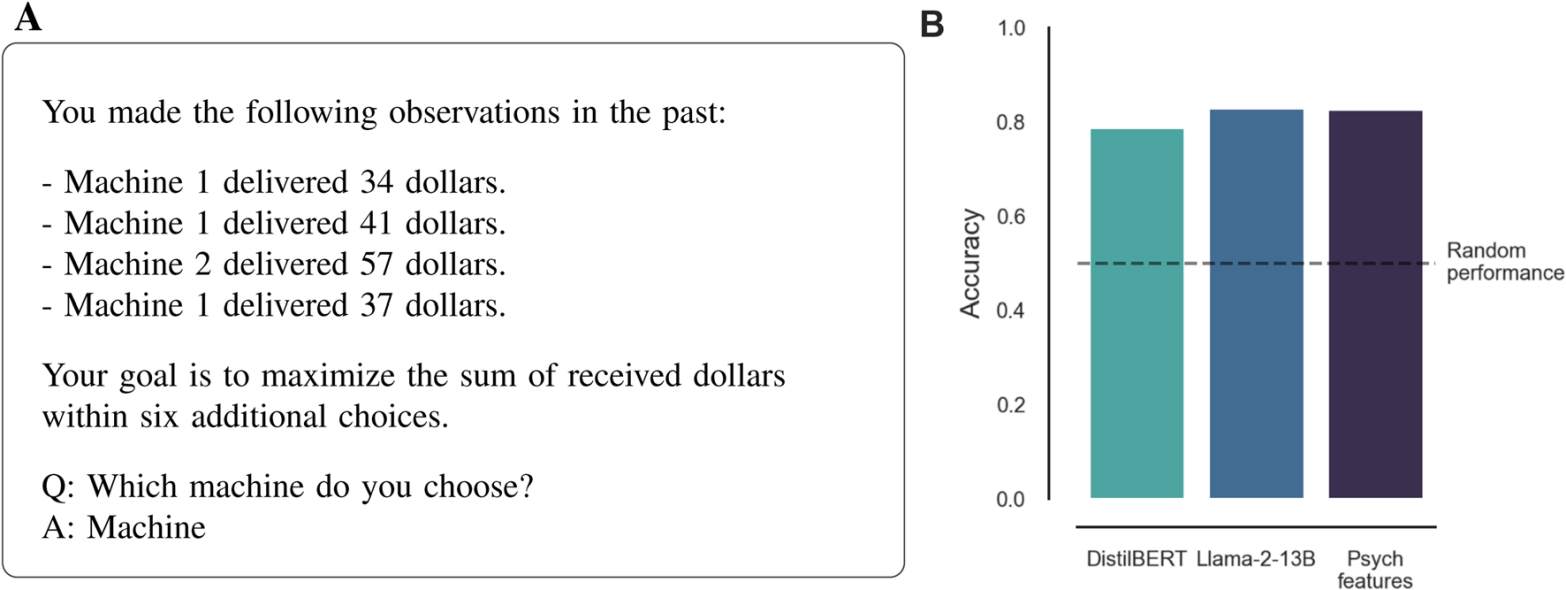
Predicting repeated choices. **A** Example prompt for the text-based version of the horizon task. **B** Accuracy of predicting human choices on the test data for different models

In line with earlier work (Binz & Schulz, 2023a; Coda-Forno et al., 2023), the model inputs, also known as prompts, are designed as follows (see also Fig. 7A). Each prompt contains information about a single game, starting with a list of previous choices and outcomes in the game. Following a brief instruction, the text continues Q: Which machine do you choose? A: Machine, missing only the number of the selected machine (1 or 2). Choices and outcomes are sequentially added to the list as the LLM interacts with the task.

Once the prompts have been loaded in a verb|pandas.DataFrame, the same out-of-sample testing pipeline used in the previous section on health judgments can be applied. The only change necessary is to replace |sklearn|’s |RidgeCV| with |LogisticRegressionCV| to account for the binary criterion (i.e., machine 1 or 2). Analogous to |RidgeCV|, |LogisticRegressionCV| also performs L2-regularization with automatic regularization parameter tuning. For the sake of brevity, we omit this code from the code block.

With the model ready, it can be fitted using the features extracted from the |’distilbert-base-uncased’| model using a training portion of the data. The resulting model achieved a considerable accuracy of $78.9\%$ on the test set. However, this performance can be improved using larger models. This time, we consider decoder models, which, based on currently available models, are better suited for the task at hand in two ways. First, current decoder models are larger in size than encoder models and can thus potentially capture more sophisticated reasoning (Brown et al., 2020). Second, their design may better match the sequential nature of the task, which mirrors the next-token prediction setting.

Fortunately, the Hugging Face interface makes it possible to use some of the most advanced models with only minor code modifications. In line with the goal of sharing reproducible code that can run in a Google Colab environment, we use a smaller Llama 2 model in the analysis example (Touvron et al., 2023). Llama 2 is a family of large-scale decoder models developed by Meta that are available in different sizes, training stages, and formats through the Hugging Face Hub. The specific model used in the example is |’TheBloke/Llama-2-13B-GPTQ’|, which has been created by quantizing the 13 billion parameter model to 4 bits to reduce its RAM footprint without a significant loss in accuracy (see, e.g., Frantar et al., 2022). With 13 billion parameters, the model is two orders of magnitude larger than the |’distilbert-base-uncased’| model used thus far.

A few additional minor modifications are needed to use the Llama 2 model. First, because ’TheBloke/Llama-2-13B-GPTQ’ is a different class of model, that is, a decoder causal language model, there is no "[CLS]" token that could be used to stand in for the entire input. Instead, in causal language models, the most important token to generate predictions is the final token in the input, that is, the token "machine", which is the only token whose contextualized embeddings are determined by those of all other tokens. The hidden state for the final token in the sequence can be extracted using last_hidden_state[:,-1]. Second, loading this model requires the more specific AutoModelForCausalLM.from_pretrained() method as opposed to the generic AutoModel.from_pretrained() method. Within the function, device_map="auto", which is available only for certain larger models, is used to allocate the model automatically to GPU or CPU resources, making the explicit casting of tensors via |.to(device)| obsolete, and |revision=’main’| is used to indicate the specific model branch. (See https://huggingface.co/TheBloke/Llama-2-13B-GPTQ for the list of options on the corresponding model card.)

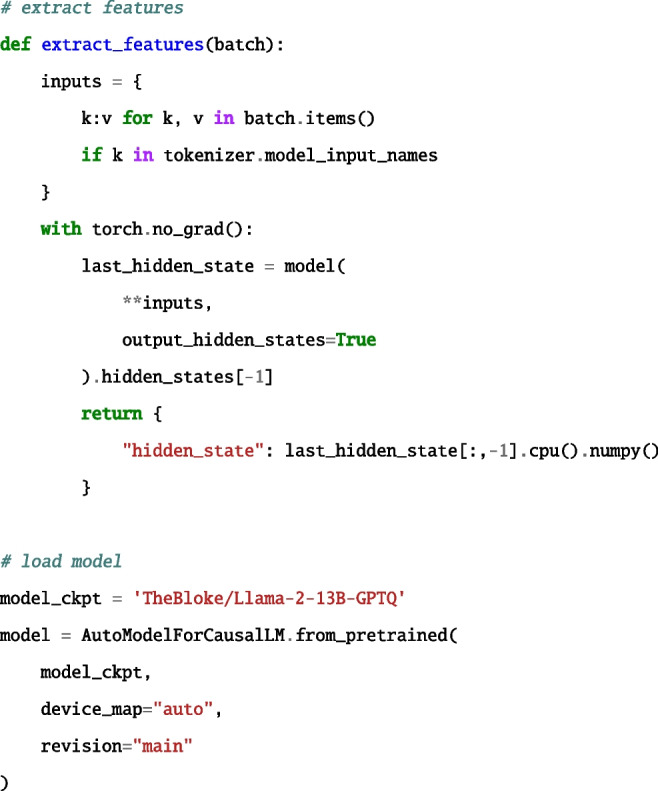



Using the 13B Llama 2 model with these settings required only around 16 GB GPU memory, implying that it can be replicated on many standard personal computers. Predicting repeated choice using the Llama 2 features resulted in a substantial increase in accuracy over the DistilBERT model to $82.9\%$ (see Fig. 7B). This performance is on a par with that of a psychological model using handcrafted features such as the difference in estimated rewards or the difference in uncertainty, as described by Gershman (2018); Binz & Schulz (2022), which achieved a test accuracy of $82.6\%$.

Overall, the results presented in this section demonstrate that LLMs can produce feature vectors that make it possible to predict complex decision-making behavior at a level comparable with that of current psychological models. Given that considerably larger models are available and that the model in the example may have suffered from quantization, it is plausible that the ceiling for state-of-the-art LLMs is considerably higher. With such strong performances, these results hint at behavioral applications of LLMs that go beyond predicting observed data, such as investigating how changes to the experimental stimuli or instructions may impact participant behavior. Such changes may be used, for instance, as in-silico pilot studies to inform future experimental designs, or to test specific hypotheses that were not testable with the original experimental data.

## Fine-tuning

Feature extraction can be a powerful approach for predicting human judgments and behavior, especially when labeled task-specific data are scarce or if the researcher does not have access to strong GPUs. However, as the extracted features are domain-general, they may not be optimal for all tasks. An alternative approach is fine-tuning, sometimes referred to as *transfer learning*. During fine-tuning, all model weights are typically optimized for the given task (for an alternative strategy, see, e.g., Hu et al., 2022).

This section again uses data from Aka & Bhatia (2022), as illustrated in Table 2. See github.com/Zak-Hussain/LLM4BeSci.git to run this example in a Google Colab environment. So as not to repeat code, we begin with |dat| already tokenized, and with the device already set to the GPU. The code starts by setting torch.manual_seed(42). This helps to ensure the reproducibility of stochastic processes such as stochastic gradient descent and its variants, including the ’adamw_torch’ optimizer used for fine-tuning the model.

The data are again split into a training and a test set. Because the plan is to use the Hugging Face ecosystem downstream as well, the data are kept in the native Hugging Face format using the inbuilt datasets.Dataset.train_test_split() method. Mirroring |sklearn|’s train_test_split(), a test set size and "random state" can be set, this time with test_size and |seed| as arguments. The method returns the split data sets in the form of a datasets.DatasetDict object, which behaves much like a regular Python dictionary. Technically, it inherits from |dict|, and has the keys |’train’| and |’test’| that map to the respective data sets.

Next, |’distilbert-base-uncased’| is initialized as the model_ckpt, but this time with transformers.AutoModelForSequenceClassification, as opposed to the basic |tranformers.AutoModel| from earlier. This loads the model with a "classification head" attached. Despite its name, this head can also be used for regression tasks such as the one at hand by specifying num_labels=1 as an argument in the from_pretrained() method.

Having initialized the model, it is now time to set up the training loop with a |transformers|’ TrainingArguments object. This involves specifying the name of the output directory where model predictions and checkpoints will be saved (output_dir), the batch size for training and evaluation (per_device_train_batch_size, per_device_eval_batch_size), and the frequency with which training and evaluation metrics are recorded (logging_strategy, evaluation_strategy). Because neural networks usually need to be trained for multiple iterations over the training set, the number of iterations, also called *epochs*, can be specified with num_train_epochs. It is important to note that more advanced strategies exist to automatically halt training when certain test-performance-optimizing heuristics are triggered (e.g., *early stopping*). However, for the purposes of this tutorial, we stick to manually specify num_train_epochs.

An evaluation function, compute_metrics(), is defined to evaluate model performance with metrics other than the model’s loss, which is automatically logged. This takes a |transformers.EvalPrediction| as input, which can be unpacked into the model’s predictions of the health perception ratings (|preds|) and the actual ratings (|labels|). An object to compute the explained variance ($R^2$) is then loaded using Hugging Face’s |evaluate| library, using evaluate.load("r_squared"), and the $R^2$ is computed with the object’s |compute()| method using the |preds| and |labels|.

Training is carried out by the transformers.Trainer. In this case, the trainer is initialized with the model (|model=model|), the data set to be used for training the model (train_dataset=dat[’train’]), the data set to be used for evaluating the model (eval_dataset=dat[’test’]), and the function to evaluate the model’s performance on the test set (compute_metrics=compute_metrics). Model training is then initiated by running |trainer.train()|.

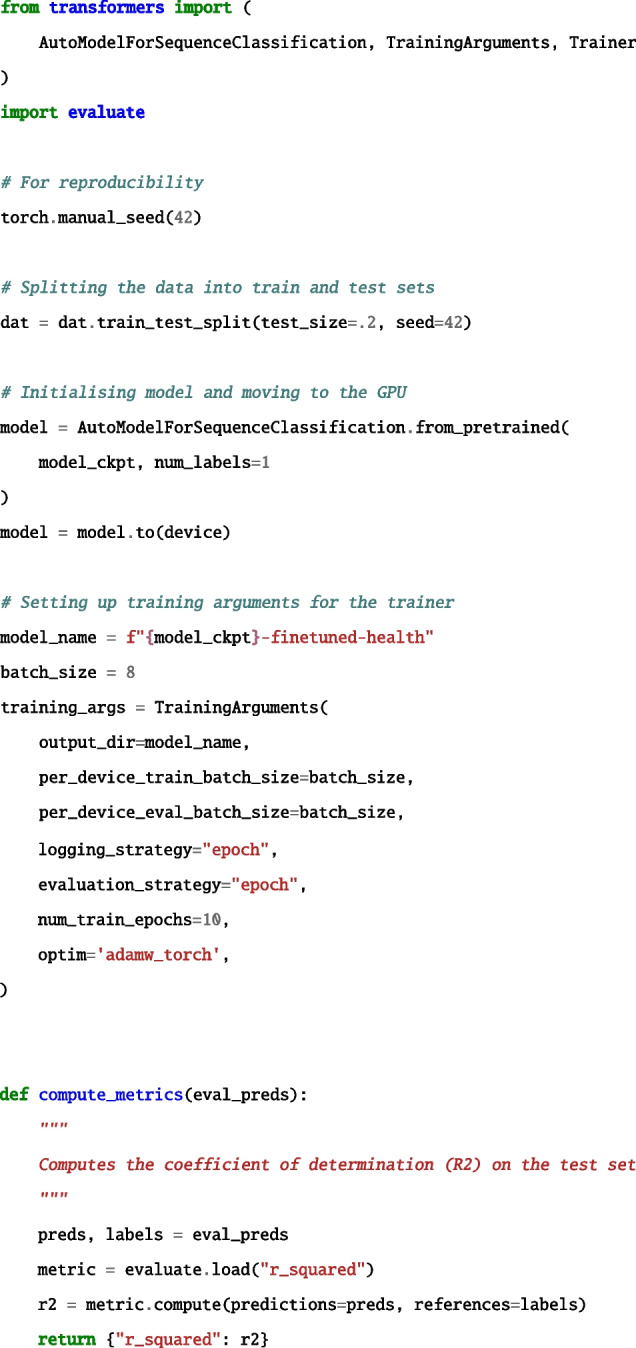

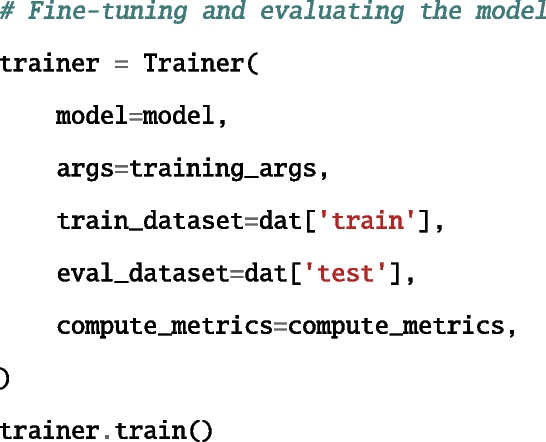





In our example, after ten epochs, test performance plateaued at around $R^2=.50$. This is slightly below the $R^2=.54$ achieved using feature extraction. As before, repeated train–test splits would need to be run using, for instance, (repeated) k-fold cross-validation, to achieve a more reliable test performance estimate. For the sake of brevity, the code for k-fold cross-validation is not included in this tutorial; however, the pattern of results remains the same. Thus, using our approach, feature extraction outperforms fine-tuning when it comes to predicting health perceptions.

This result highlights that fine-tuning may not always be the dominant choice when it comes to using LLMs for predicting human judgments and behavior. Factors such as data quantity and quality as well as the availability of computational resources will play a large role in determining which approach makes the most sense. It is also worth noting that a host of fine-tuned models are available for download from the Hugging Face Hub. As such, depending on the task at hand, the researcher may find that fine-tuned models for specific tasks or domains are already available at huggingface.co/models. Of course, the data used for fine-tuning may still differ considerably from the data that the researcher wishes to apply the fine-tuned model to. As such, it is always important to compare fine-tuned models to their pre-trained alternatives.

## Text generation

Perhaps one of the major features contributing to the proliferation of LLMs is their ability to generate convincing, human-like text in response to prompts. In behavioral science, this capacity has made it possible to perform a vast battery of in-silico behavioral experiments (e.g., Yax et al., 2023; Binz & Schulz, 2023b): As long as the experiment can be converted into a text-based format – setting multimodal models aside for present purposes – the model can "participate" in it.

Following Yax et al. (2023), this section draws on the example of the cognitive reflection test (CRT) to demonstrate how this can be done (Frederick, 2005). The three-question CRT is designed to measure a person’s ability to inhibit intuitive but incorrect responses in favour of deliberation. Here, we focus on how researchers can run experiments on LLMs in a *replicable* manner on their own hardware or using freely available cloud computing resources. See github.com/Zak-Hussain/LLM4BeSci.git to run this example in a Google Colab environment.

We again use a quantized 13 billion parameter version of Llama 2 (Touvron et al., 2023). As for predicting repeated choice, despite the vast increase in model size, the code for running the model uses some familiar Hugging Face components: a |transformers.pipeline| is used, the model_ckpt is defined and passed as an argument to |model| and |tokenizer| when initializing the pipeline. Likewise, device_map=’auto’ is used for efficient upscaling to larger models.

However, as the goal is to have the model respond to the CRT questions with text, there are two main differences from predicting repeated choice. First, a transformers.pipeline for ’text_generation’ is used, which gets the model to generate responses with minimal code. Second, a chat-based version of Llama 2, ’TheBloke/Llama-2-13B-chat-GPTQ’, is employed (TheBloke, 2022). This model version has been further trained with techniques such as supervised fine-tuning and reinforcement learning from human feedback to align it more closely with the preferences of humans who wish to use it as an AI assistant (Touvron et al., 2023).

The final arguments in the pipeline are specifically to do with text generation. max_new_tokens=512 means that the model can produce a maximum of 512 tokens in response to the prompt. do_sample=False prevents the model from performing random sampling from the softmax output distribution over its vocabulary. This forces the model to employ a strategy known as *greedy search decoding*, whereby the model output at each timestep is simply the token with the highest probability in the output distribution. This can be contrasted with text-generation techniques involving sampling, which seek to improve the diversity of model outputs by adding some randomness to the generation process. However, this added diversity can come at the expense of coherence and reproducibility (Tunstall et al., 2022).

The CRT comprises three questions, which are assigned to |prompt|. In order to create a more realistic experimental context for the model, the code uses the Llama 2 chat-specific prompting template recommended on the |’TheBloke/Llama-2-13B-chat-GPTQ’| model card page (TheBloke, 2022):

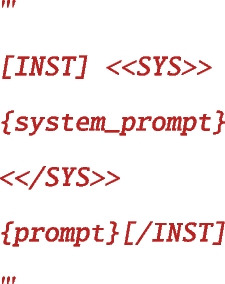



In this case, the |system prompt|, which is the broader context given to the model to help guide it, is a general description of the psychological study along with the instructions for participants, whereas the |prompt| contains the CRT questions. Both together form the prompt_template.

Generating the output is then as straightforward as passing the prompt_template to |generator|, which returns the output of the model as a list. The generated tokens can be accessed with the zero index, which returns a dictionary with a single key: ’generated_text’. Accessing the key’s value returns a string composed of the text generated by the model with the input (prompt_template) prepended. In order to return only the output generated, the string is sliced such that the printed output begins after the last character of the prompt_template.

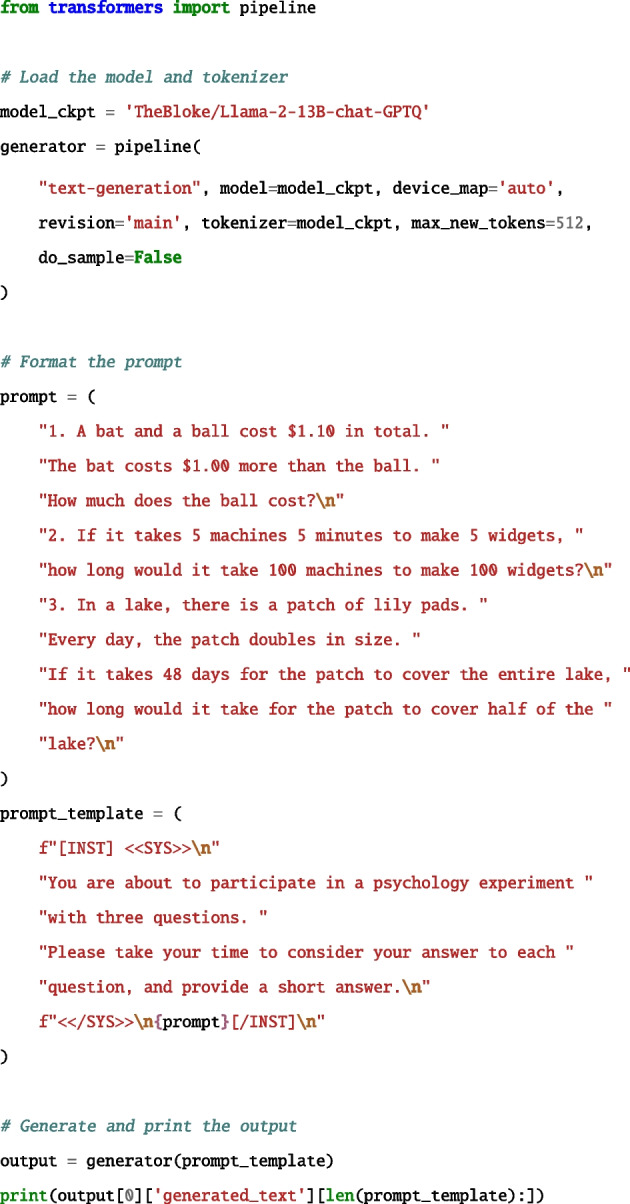



The model output is the following. It answers two out of the three questions correctly.

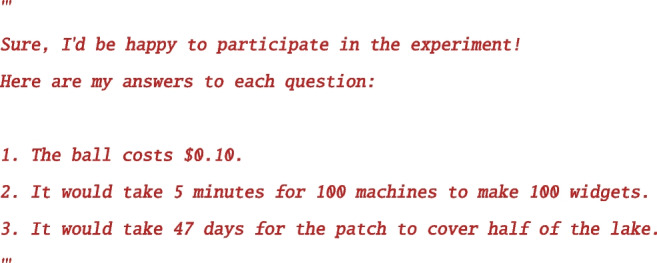



On average, human participants answer between 1 and 2 questions correctly, suggesting that LLMs can answer problems from the CRT at or above human level. However, one should be careful not to draw strong conclusions about the models’ reasoning capabilities from these results. It is possible that examples of the CRT are in Llama 2’s pre-training set, with (in-)correct answers included. Similar to findings on CRT pre-exposure among humans (Haigh, 2016), this may inflate the model’s performance and conclusions about its ability to reason (Mitchell, 2023).

**Table 3:** Three examples from Crossley et al. (2023)’s CLEAR corpus containing text excerpts leveled for 3rd to 12th grade readers and teachers’ readability scores

Excerpt	BT_easiness
An honest and poor old woman was...	-0.05
Our plate illustrates the residence of...	-2.98
Just as wildebeest are the main grazers...	-2.46

The main purpose of this example is to show that open-access LLMs such as Llama 2 can be run on freely available hardware and used as stand-in "participants" in behavioral experiments, with the potential to generate insights about the experiment, the model, and perhaps human psychology as well.

## Token probability and perplexity extraction

This tutorial has introduced three common LLM use cases: feature extraction, fine-tuning, and text generation. Although these are perhaps more well-known use cases, they are not exhaustive. For instance, an additional use of LLMs is to deploy them on the very tasks for which they were pre-trained; namely, to assign probabilities to tokens. During training, the model is incentivized to assign high probabilities to tokens and token sequences that are common in the training data and vice versa for those that are uncommon. As a result, the probabilities produced by a trained model can be used to detect text sequences that (the model has learned) are uncommon. Measures based on token and token sequence probabilities have thus been used to, for instance, investigate how language models capture grammatical gender (An et al., 2019) and to predict human reading times (e.g., Merkx & Frank, 2020).

The present example demonstrates how the log probabilities extracted from GPT-2 can be used to predict teachers’ text readability ratings. Specifically, these are teachers’ ratings of how difficult student readers would find certain text excerpts, obtained from the CommonLit Ease of Readability (CLEAR) corpus (Table 3; Crossley et al., 2023).

A common metric to evaluate the probability of a sequence is called *perplexity* (Jelinek et al., 1977). Given a sequence of tokens $X=(x_{0}, x_{1}, ..., x_{t})$, perplexity can be defined as

$$ exp\left( -\frac{1}{t}\sum _{i=1}^{t}\log p(x_{i} \vert x_{<i})\right) $$

with $p(x_{i} \vert x_{<i}){ beingtheprobabilityassignedtothe}i$-th token in the sequence given the preceding tokens. Perplexity is thus the inverse geometric mean of sequentially produced token probabilities. As perplexity assumes sequentially produced tokens, it is not well-defined for masked language modeling. In our example, we therefore rely on GPT-2, which is a decoder model trained using causal language modeling and a direct precursor to today’s GPT-4.

The code begins by reading the |’Exerpt’| and ’BT_easiness’ columns of the CLEAR corpus into a pandas.DataFrame. It then loads a perplexity metric object from the |evaluate| library’s |load| method. This object has a convenient |compute| method, which allows the researcher to specify from which model they wish to compute the perplexity (model_id=’openai-community/gpt2’ in this case), the text sequences to compute perplexity on (|clear[’Excerpt’])|), the batch size (|8|). The device defaults to the GPU if a CUDA-compatible GPU is available. The method returns a dictionary containing a list of perplexity scores for each sample (|’perplexities’|) and the average of those scores (’mean_perplexity’).

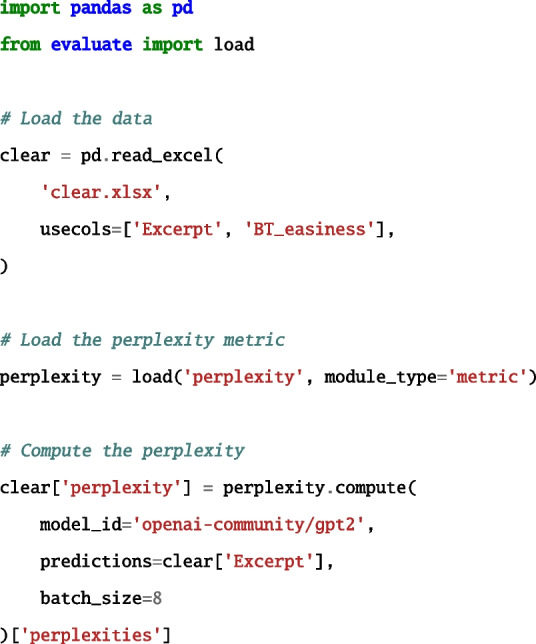



To evaluate whether perplexity can predict readability, we feed perplexity as a feature into an ordinary least squares regression. Using 1000 randomly sampled excerpts from CLEAR, the perplexity model achieves a tenfold cross-validation $R^2=.20 (\text {SD}=.05)$, implying that perplexity can account for a significant portion of text readability variance. The predictive accuracy of perplexity can be compared to an established predictor of readability, the Flesch Reading Ease (Flesch, 1948), calculated based on average sentence and word lengths. Flesch Reading Ease achieves an $R^2=.28 (\text {SD}=.04),\,{ whichisonlyslightlyhigherthantheperformanceofperplexity}.{ Moreover},\,{ combiningthetwofeaturesleadstoaconsiderableincreaseinpredictiveaccuracy}(R^2=.44,\,\text {SD}=.05$), indicating the added value of perplexity for the prediction of readability.

Compared to the other use cases presented above, token probability and perplexity measures have been less frequently employed in behavioral science research. Nevertheless, these measures show promise in behavioral research in at least two respects. First, they can be used to predict human perceptions and evaluations of text, such as the readability of study vignettes or the surprisingness of statements. Secondly, they can be employed to evaluate the likelihood of human responses to text, such as response to open-text format items.

## Open questions and future directions

This tutorial has introduced the Hugging Face ecosystem as an open-source approach to using LLMs in behavioral science. This section discusses the advantages and limitations of (open-source) language modeling in behavioral science, the broader societal-level risks posed by (open-source) LLMs, and future directions for behavioral science research with LLMs.

### Good science practices with LLMs

The goal of this tutorial has been to make the responsible use of LLMs for behavioral science more accessible. However, using LLMs *responsibly* includes following good science practices. This requires more than the ability to implement LLMs in code; it also necessitates a substantive understanding of what that code is doing and an appreciation of the complex and nuanced theoretical questions concerning LLM capabilities. We hope that through Section “A primer on transformer-based language models”, and the explanations accompanying the code in this tutorial, readers will come away with a thorough understanding of what this code is doing. Nevertheless, a few cautionary points are in order.

The first concerns hyper-parameters. Like most popular statistical libraries in behavioral science, Hugging Face libraries enable control over a vast array of hyper-parameters, and many of these hyper-parameters have default settings. Although these defaults often make code more readable, they can also lead to complacency. Against this tendency, we stress the importance of making active decisions between the universes of possible hyper-parameter settings. In cases where substantive justifications are lacking, we encourage the use of *multiverse analyses* (i.e., with different plausible hyper-parameter setups; Steegen et al., 2016), computational resources permitting.

The second concerns performance evaluation. In addition to repeated out-of-sample testing, we emphasize the importance of other evaluation and sanity-checking strategies, such as testing meaningful synthetic baseline models. This could include randomly shuffling the pairings between extracted features and labels to identify possible data leakages – a pernicious problem in machine learning more broadly (see, e.g., Kaufman et al., 2012). Alternatively, it could mean artificially constructing perfectly predictive fine-tuning features to ensure model fitting is working properly.

The third and final point is more theoretical. The recent proliferation of LLMs has revived long-running debates over whether LLMs possess capacities such as *true* "understanding" ("thinking", "reasoning", etc.; see, e.g., Mitchell & Krakauer, 2023). Although we believe that the validity of the applications described in this tutorial does not depend on whether LLMs truly possess such capacities, we anticipate that the broader conclusions drawn from scientific studies involving LLMs will often be mediated by researchers’ background beliefs concerning these questions. As such, we would like to highlight the existence of a vast scientific and philosophical literature pertaining to the (evaluation of) language model capabilities (e.g. Mitchell & Krakauer, 2023; Turing, 1950; Bender et al., 2021; Searle, 1980; Günther et al., 2019) – a literature that has by no means reached a consensus – to guard against snap judgments or uncritical default positions.

In their broader form, these words of caution do not concern LLMs alone but are part of good science practice more generally. However, the complexity, opacity, and quirkiness of neural network models can exacerbate these issues in ways that require special attention.

### Open-source LLMs and open behavioral science

Behavioral science is going through an open science revolution guided by principles such as transparency, accessibility, and sharing (Vicente-Saez & Martinez-Fuentes, 2018). Open-source and open-access language modeling frameworks such as Hugging Face are closely aligned with these principles. For instance, with Hugging Face, all analysis steps – from data preprocessing to model validation – are in principle accessible to fellow scientists wishing to better understand and perhaps reproduce what others have done. Likewise, models fine-tuned using the |transformers| library can easily be shared on the Hugging Face Hub, making it easier for researchers to build on and benefit from the work of their peers. With over 300,000 models and 60,000 datasets, Hugging Face stands as an exemplary case of the power of sharing in research and beyond.

Hugging Face also supports reproducibility. Features such as the ability to set seeds help improve the reproducibility of nondeterministic processes such as gradient descent. Likewise, because models are saved to the hard drive (instructions for locating models saved to hard drive are regularly updated at stackoverflow.com/questions/61798573/where-does-hugging-faces-transformers-save-models), the precise version of the model used for the analysis can be permanently saved for future reproductions. This stands in contrast to less open alternatives such as the OpenAI API, which, at the time of writing, does not provide the ability to access the same version of the model indefinitely into the future after model updates.

Open-source and open-access language modeling frameworks also have considerable advantages when it comes to data privacy: Because models can be saved and run locally, sensitive data can remain on the appropriate hardware, and researchers can be sure that the creators of the model will not have access to it. This is crucial in behavioral science, where ensuring the privacy of participant data is paramount from an ethical and legal perspective.

Open-source language modeling frameworks also help mitigate an important disadvantage of LLMs: their poor interpretability. Interpretability is a general problem for neural network models, whose complexity and abstract representational nature have earned them the label "black-box" models (Alishahi et al., 2019). In behavioral science, the limited interpretability of LLMs can hinder a researcher’s ability to draw strong theoretical conclusions. For instance, being able to interpret the internal states of the model presented in the section on Text Generation would help clarify whether it had simply "memorized" answers to the CRT or actually "reasoned" them through. However, a commitment to openness – in this case, transparency about the data used to train the model – could also help resolve this uncertainty by revealing whether the CRT even featured in the training data at all. In general, interpretability is worsened when researchers are not given information about important details concerning the model’s pre-training data, tokenizer, architecture, weights, and fine-tuning regimes. Of course, even when these details are known, it can remain a mystery why a model performs well on some tasks but not on others. This point is exemplified by the existence of *emergent abilities* in LLMs: abilities that arise from model upscaling, but whose arrival is incredibly difficult to predict, even for the developers who trained the model (Wei et al., 2022).

### (Open-source) LLMs and society

Although we believe that open-source and open-access language modeling has its advantages for research, making LLMs publicly accessible also comes with considerable risks. LLMs are, after all, powerful tools, and in the hands of bad actors, they could be used to do serious harm (e.g., spreading mis- and disinformation; Weidinger et al., 2022). Increasing access to LLMs will also have environmental impacts (Strubell et al., 2019), especially when more researchers have the ability not only to use these models for inference but also to train them via ecosystems such as Hugging Face.

Furthermore, it is important to be aware of the broader risks that present and future LLMs may pose to society, especially if they are poorly aligned with people’s preferences and values (Bockting et al., 2023; Russell, 2019). Concerns such as these have motivated research programs into AI alignment from leading AI companies (Leike & Sutskever, 2023; Leike et al., 2018). They have also led to the use of more explicit human behavioral data for fine-tuning LLMs (e.g., via explicit human feedback on model outputs) to achieve closer alignment with human preferences. As has been pointed out (Irving & Askell, 2019; Russell, 2019), this endeavor presents a unique opportunity for behavioral scientists, who of course have expertise in collecting high-quality human data.

### Future direction for LLMs and behavioral science

This insight points to an interesting future direction at the intersection of behavioral science and language modeling. Although this tutorial has focused on how LLMs can be used as tools for behavioral science research (LLMs $\rightarrow$ behavioral science), an inverse relationship also holds promise: the use of behavioral science methods to build more interpretable, human-aligned LLMs (behavioral science $\rightarrow$ LLMs). For instance, as foreshadowed in the section on Text Generation, behavioral experiments can be run on LLMs to provide greater insight into the models’ capabilities (e.g., Binz & Schulz, 2023b; Yax et al., 2023). Likewise, interpretability techniques such as probing can be combined with psycholinguistic methods to assess how aligned the internal representations of LLMs are to people’s semantic representations (Cassani et al., 2023; Sucholutsky et al., 2023; Aeschbach et al., 2024). This relationship is mutually reinforcing (behavioral science $\rightarrow { LLMs}\rightarrow$ behavioral science): LLMs that are more aligned with people may also serve as more plausible and predictive psychological models (e.g., Binz & Schulz, 2023a), and LLMs that are more interpretable may allow for deeper theoretical insights – into their psychological plausibility, but also into human cognition and behavior itself.

## Conclusion

LLMs hold immense promise for behavioral science and open-source frameworks play a crucial role in promoting transparency, reproducibility, and data protection. This tutorial has provided a practical overview of using the open-source Hugging Face ecosystem, covering feature extraction, fine-tuning, and text generation, in order to empower researchers to harness the potential of LLMs for behavioral applications. While acknowledging the current limitations of this approach, we hope that these efforts can help catalyze LLM utilization and offer novel insights and opportunities for future research in behavioral science.

## Open practices statement

The data used in all sections are available at github.com/Zak-Hussain/LLM4BeSci.git. Aside from the specific results for *Cohere* (Cohere-embed-english-v3.0) and *ada* (text-embedding-ada-002) – these models are behind a paywall – all results can be reproduced using freely available software. None of the analyses were preregistered, as they are presented for demonstration purposes only.

## Data Availability

The datasets analyzed for the current tutorial are available in the *LLM4BeSci* repository, https://github.com/Zak-Hussain/LLM4BeSci.git.

## Code availability

The code used for the current tutorial is available in the *LLM4BeSci* repository, https://github.com/Zak-Hussain/LLM4BeSci.git.
